# Unveiling non‐small cell lung cancer‐specific circulating Treg subtype: A paradigm shift in single‐cell immunology and its clinical implications

**DOI:** 10.1002/ctm2.70724

**Published:** 2026-06-22

**Authors:** Zeyu Wang, Bin Li, Xueyu Dai

**Affiliations:** ^1^ Center for Immune‐Related Diseases at Shanghai Institute of Immunology, Department of Immunology and Microbiology Shanghai Jiao Tong University School of Medicine Shanghai China; ^2^ Department of Thoracic Surgery, Shanghai Pulmonary Hospital School of Medicine, Tongji University Shanghai China

1

Single‐cell RNA sequencing (scRNA‐seq) has profoundly transformed our understanding of immune‐cell heterogeneity, intercellular communication, and disease‐associated transcriptional programs. Through unbiased transcriptomic profiling, numerous immune cell subtypes and states have been described, offering unprecedented opportunities to dissect disease mechanisms and identify novel diagnostic or therapeutic targets.^1^ However, the rapid expansion of cell annotations and cell identity marker gene panels (ciMGPs) has also exposed a critical bottleneck: while new immune subsets are continuously proposed, their validation, standardisation, and clinical translatability remain insufficiently addressed.^2^ Selecting disease‐specific immune cell subtypes that are biologically meaningful, technically robust, and clinically measurable is now one of the central challenges in translational single‐cell immunology.

To address this unmet need, Wang and colleagues previously proposed the regional overlap expression rate (rOER) as a quantitative metric to evaluate the specificity of ciMGPs in pulmonary single‐cell datasets.^3^ By measuring the overlap of marker gene expression across pathological conditions, rOER provided an initial framework to classify ciMGPs into distinct specificity categories. Yet, whether this concept could be extended to dynamically changing circulating immune cells—particularly across temporal, anatomical, and disease contexts—remained unresolved.

In their recent study, Liu et al. significantly advance this framework by developing and applying an expanded rOER‐based system to identify and validate disease‐specific immune cell subtypes in non‐small cell lung cancer (NSCLC).^4^ This work represents both a methodological and conceptual leap forward. By leveraging principles derived from box‐plot statistics, the authors establish a rigorous, data‐driven approach to quantify ciMGP specificity, reproducibility, and robustness across samples, organs, and diseases. Importantly, this system moves beyond arbitrary fold‐change thresholds and subjective annotations, categorising ciMGPs into subset‐specific (ss), subset‐associated (sa), and subset‐reference (sr) groups. In a field often plagued by batch effects, gene dropout, and inconsistent nomenclature, the rOER framework directly confronts the long‐standing “identity crisis” of single‐cell biology. Using this strategy, the authors identify and validate a distinct circulating regulatory T (Treg) cell subtype, termed Treg^fci^, defined by the ss‐ciMGP co‐expression of *FOXP3*, *CTLA4*, and *IL2RA*. Treg^fci^ displays striking disease‐, organ‐, and time‐specific characteristics: it is markedly enriched in pre‐operative peripheral blood and NSCLC tissues, declines or disappears following tumour resection, and localises spatially to the tumour–normal interface. Notably, its prevalence is significantly higher in NSCLC than in other chronic lung diseases or most extrapulmonary malignancies, underscoring its disease specificity.

Mechanistically, the study identifies the transcription factor ETS1 as a central regulator of Treg^fci^ identity and function. ETS1 is consistently upregulated in Treg^fci^ across circulation and tumour tissues, and functional experiments demonstrate that ETS1 promotes Treg^fci^ migration from the bloodstream into tumour sites, likely in response to inflammatory cues. Moreover, ETS1‐dependent regulation extends beyond migration, influencing mitochondrial metabolism, intracellular organelle interactions, and cytokine production. Downregulation of Treg^fci^ ciMGPs or ETS1 impairs cell viability and proliferative capacity while reshaping cytokine profiles, highlighting Treg^fci^ as a biologically relevant Treg subset in NSCLC progression (Figure 1).

**FIGURE 1:**
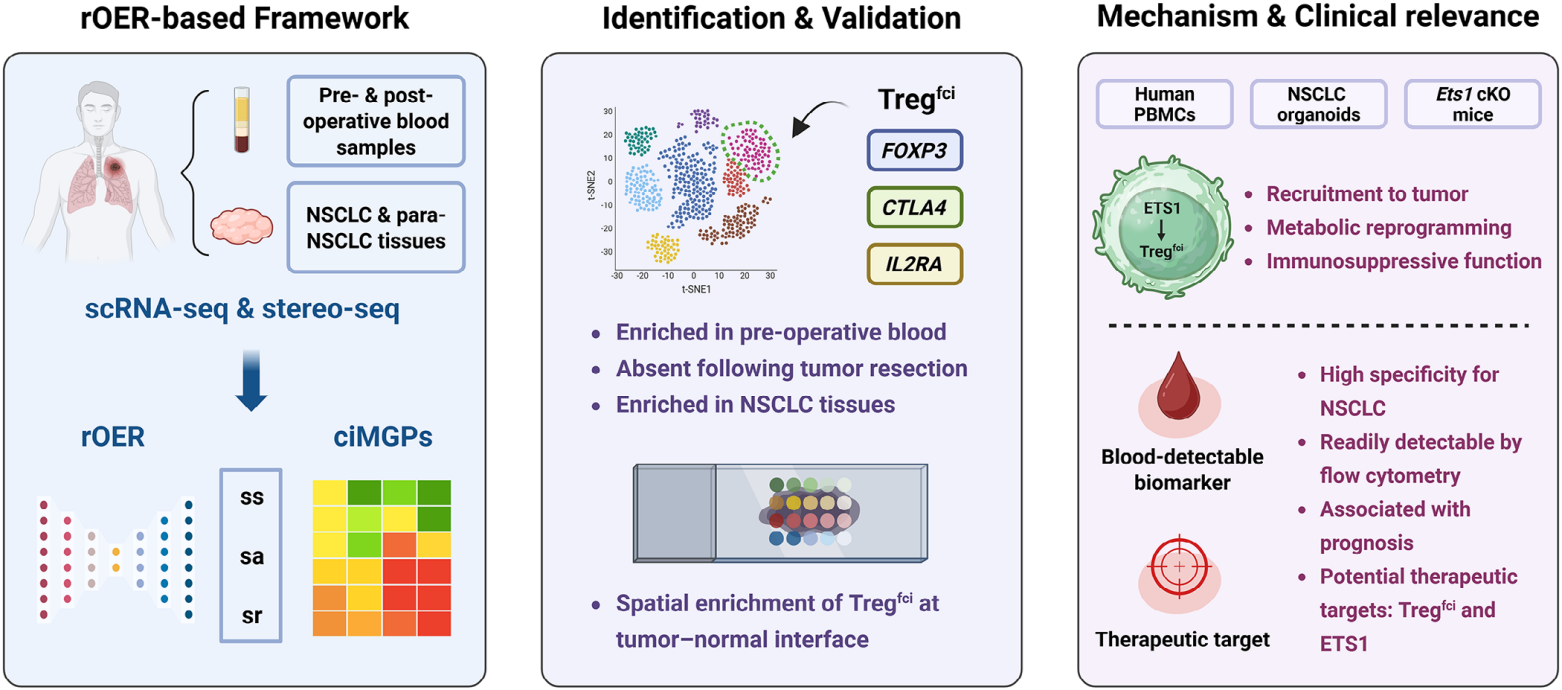
Unveiling non‐small cell lung cancer (NSCLC)‐specific circulating Treg subtype using a regional overlap expression rate (rOER)‐based framework. Integrated single‐cell and spatial transcriptomic analyses identified a novel NSCLC‐specific circulating Treg subtype, enriched in pre‐operative blood and NSCLC tissues, reduced after tumour resection, and localised to the tumour–normal interface. ETS1 regulates Treg^fci^ tumour recruitment, metabolic reprogramming, and immunosuppressive function. Treg^fci^ is detectable in peripheral blood, associated with poor prognosis, and represents a potential immunotherapeutic target together with ETS1.

Single‐cell transcriptomic profiling of peripheral blood immune cells has become a powerful approach for deciphering systemic disease responses, identifying disease‐specific biomarkers, and characterising immune heterogeneity.^5^ Reyes et al. demonstrated that distinct peripheral blood immune cell states correlate with septic status and identified a sepsis‐specific monocyte population, termed MS1, characterized by CD14 expression and elevated levels of *RETN*, *ALOX5AP* and *IL1R2*.^6^ Through rigorous validation across independent RNA‐seq datasets and previously reported classifiers, the authors further defined a clinically tractable surface marker panel—low HLA‐DR and high IL1R2—enabling cytometric identification of the MS1 state. This study illustrates how ciMGP‐defined immune cell states can be translated into clinically measurable biomarker panels. Notably, such translational success depends critically on the specificity and robustness of ciMGPs, highlighting a central challenge for the clinical application of peripheral blood scRNA‐seq. In the present study, Liu et al. address this issue by applying an rOER‐based framework to systematically quantify the disease‐, organ‐, and time‐specificity of ciMGPs. Using this approach, they demonstrate that Treg^fci^ exhibits high NSCLC specificity. Moreover, the ss‐ciMGP signature defining Treg^fci^ is readily detectable by flow cytometry and immunostaining, substantially enhancing its clinical translational potential.

The complexity of Treg cell development, function and metabolism presents challenges to the precise definition of subset identities.^7^ The existence of multiple Treg subsets across tissues and microenvironments further complicates this landscape.^8^ In the tumour microenvironment (TME), the perturbation of Treg cells—manifested by reduced FOXP3 expression, enhanced production of pro‐inflammatory cytokines, and metabolic reprogramming—can critically contribute to antitumor immune responses.^9^ ETS1 plays a central role in maintaining Treg lineage stability by promoting FOXP3 transcription and preserving the suppressive Treg phenotype through direct binding to conserved promoter and enhancer elements of the *FOXP3* locus.^10^ Loss of ETS1 leads to diminished FOXP3 expression, destabilisation of the Treg lineage, and impaired peripheral tolerance. Consistent with these established functions, Liu et al. report that NSCLC tumours exhibit slower growth and a reduced proportion of Treg^fci^ in Treg‐specific *Ets1* conditional knockout mice. Moreover, pharmacological inhibition of ETS1 in human PBMCs and NSCLC organoids confirms its regulatory role in reshaping Treg^fci^ transcriptomic and metabolic programs. Collectively, these findings identify Treg^fci^ and its key regulator ETS1 as potential therapeutic targets and provide new insights into Treg‐mediated immune regulation within the TME.

In summary, Liu et al. deliver a landmark study that integrates computational rigour, spatial transcriptomics, functional perturbation, and clinical correlation. By introducing an extensible rOER framework and elucidating the biology of the ETS1‐driven Treg^fci^ subtype, this work provides a compelling blueprint for translating single‐cell discoveries into clinically meaningful biomarkers and targets. Looking ahead, prospective cohort studies will be essential to determine whether dynamic changes in circulating Treg^fci^ can predict response to neoadjuvant therapy, signal early recurrence, or guide treatment stratification in NSCLC. Integration of the Treg^fci^ signature with circulating tumour DNA or protein biomarkers may further enable the development of powerful composite liquid biopsy platforms for precision oncology.

## CONFLICT OF INTEREST STATEMENT

The authors declare no conflict of interest.
